# Beyond drug‐induced alteration of glutamate homeostasis, astrocytes may contribute to dopamine‐dependent intrastriatal functional shifts that underlie the development of drug addiction: A working hypothesis

**DOI:** 10.1111/ejn.14416

**Published:** 2019-05-14

**Authors:** Maxime Fouyssac, David Belin

**Affiliations:** ^1^ Department of Psychology University of Cambridge Cambridge UK

**Keywords:** addiction, astrocytes, dopamine, glutamate, habits, relapse, striatum, tripartite synapse

## Abstract

The transition from recreational drug use to compulsive drug‐seeking habits, the hallmark of addiction, has been shown to depend on a shift in the locus of control over behaviour from the ventral to the dorsolateral striatum. This process has hitherto been considered to depend on the aberrant engagement of dopamine‐dependent plasticity processes within neuronal networks. However, exposure to drugs of abuse also triggers cellular and molecular adaptations in astrocytes within the striatum which could potentially contribute to the intrastriatal transitions observed during the development of drug addiction. Pharmacological interventions aiming to restore the astrocytic mechanisms responsible for maintaining homeostatic glutamate concentrations in the nucleus accumbens, that are altered by chronic exposure to addictive drugs, abolish the propensity to relapse in both preclinical and, to a lesser extent, clinical studies. Exposure to drugs of abuse also alters the function of astrocytes in the dorsolateral striatum, wherein dopaminergic mechanisms control drug‐seeking habits, associated compulsivity and relapse. This suggests that drug‐induced alterations in the glutamatergic homeostasis maintained by astrocytes throughout the entire striatum may interact with dopaminergic mechanisms to promote aberrant plasticity processes that contribute to the maintenance of maladaptive drug‐seeking habits. Capitalising on growing evidence that astrocytes play a fundamental regulatory role in glutamate and dopamine transmission in the striatum, we present an innovative model of a quadripartite synaptic microenvironment within which astrocytes channel functional interactions between the dopaminergic and glutamatergic systems that may represent the primary striatal functional unit that undergoes drug‐induced adaptations eventually leading to addiction.

## Graphical abstract

Astrocytes may channel functional interactions between the dopaminergic and glutamatergic systems within a quadripartite synaptic microenvironment which may represent the primary striatal functional unit that undergoes drug‐induced adaptations that eventually lead to addiction.

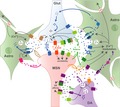

**Abbreviations**
A1: adenosine 1 receptor
A2A: adenosine 2A receptor
Acb: nucleus accumbens
AcbC: nucleus accumbens core
AcbS: nucleus accumbens shell
Adeno: adenosine
aDLS: anterior dorsolateral striatum
A‐O: action‐outcome
Astro: astrocyte
ATP: adenosine triphosphate
B‐end: beta‐endorphin
Ca^2+^: calcium
CB1: cannabinoid receptor 1
CeN: central nucleus amygdala
CNS: central nervous system
COMT: catechol‐o‐methyltransferase
CS: conditioned stimulus
D1: dopamine receptor D1
D2: dopamine receptor D2
DA: dopamine
DAT: dopamine active transporter
DLS: dorsolateral striatum
DOR: δ‐opioid receptor
DR: dopamine receptor
Dyn: dynorphin
eCBs: endocannabinoids
Enk: enkephalin
GABA: gamma‐aminobutyric acid
GLAST: glutamate aspartate transporter
GLT‐1: glutamate transporter 1
Glut: glutamate
KOR: κ‐opioid receptor
LgA: long access
LTD: long‐term depression
LTP: long‐term potentiation
MAO: monoamine oxydase
mGluR: metabotropic glutamate receptor
MOR: μ‐opioid receptor
MSN(s): medium spiny neuron(s)
NAC: N‐acetylcysteine
pDMS: posterior dorsomedial striatum
PFC: prefrontal cortex
PL: prelimbic cortex
ShA: short access
VTA: ventral tegmental area
xCT: cystine‐glutamate exchanger

## 1 INTRODUCTION

Drugs of abuse induce a broad spectrum of central and systemic effects that vary depending on their mechanisms of action (Nestler, 2005). Despite differences in their neurobiological targets and associated subjective effects, addictive drugs share a common neurochemical mechanism, in that they all increase the extracellular concentration of dopamine in the striatum (Di Chiara & Imperato, 1988).

The striatum is the main entry point of the basal ganglia, receiving glutamatergic inputs from the cortex following a topographical organisation in parallel loops (Haber, 2016). Broadly, therefore the striatum is involved in motor control, motor learning, instrumental conditioning, executive functions, emotion and motivation (Lanciego, Luquin, & Obeso, 2012; Ward, Seri, & Cavanna, 2013). It is a cytoarchitecturally heterogeneous structure (Kreitzer, 2009) composed of projecting GABAergic neurons, i.e. medium spiny neurons (MSNs), that form the first GABAergic node of the cortico‐striato‐pallido‐thalamocoritcal loops (Kita, 1993), as well as GABAergic (fast spiking) and cholinergic (tonically activated) interneurons. MSNs are further subdivided functionally by the dopamine receptor subtype they express (D1‐like, D2‐like or both), and/or their efferent target.

This cytoarchitectural landscape is highly contrasted between the ventral and dorsal territories of the striatum, which have different cortical and limbic inputs and functionally distinct roles (Voorn, Vanderschuren, Groenewegen, Robbins, & Pennartz, 2004).

The nucleus accumbens (Acb), the most ventral territory of the striatum and long considered a “limbic‐motor interface” (Mogenson, Jones, & Yim, 1980), is part of the mesolimbic pathway, also known as the “reward pathway.” The mesolimbic pathway consists of midbrain dopaminergic neurons in the ventral tegmental area (VTA) that project to the shell (AcbS) and core (AcbC) sub‐territories of the nucleus accumbens, as well as the amygdala and the hippocampus (Ikemoto, 2010).

Dopaminergic neurons within the mesolimbic pathway are phasically activated in response to rewarding outcomes. Importantly, if the reward is preceded by a conditioned stimulus (CS) that reliably predicts the outcome, the firing of these neurons shifts to the CS. Consequently, the activation of mesolimbic dopamine neurons has been suggested to convey a prediction error signal about the value of future outcomes (Schultz, 2010). Within the accumbens, the AcbS has been shown to contribute to the reinforcing properties of addictive drugs while the AcbC is involved, through its inputs from the orbitofrontal cortex and the amygdala, in bridging pavlovian influences over instrumental responding (Everitt & Robbins, 2005; Schultz, 1998, 2000; Wise, 2004). Consequently, the Acb has been suggested as an integrative region, encoding the incentive motivational properties of stimuli (Cardinal, Pennicott, Sugathapala, Robbins, & Everitt, 2001; Flagel et al., 2011) and the valence of outcomes. These are integrated with bodily information concerning current and past internal states, as well as instrumental responses mediated by the medial or lateral dorsal territories of the striatum on which action‐outcome or stimulus response associations depend or stimulus response associations respectively (Shiflett, Brown, & Balleine, 2010; Thorn, Atallah, Howe, & Graybiel, 2010), to orchestrate adaptive motivated behaviours.

These complementary roles of the striatum have been formalised into a putative model, wherein the Acb is considered the “Critic,” whereas the dorsal striatum is considered the “Actor,” mapping relationships between states and action propensities (Le Masurier, Zetterstrom, Cowen, & Sharp, 2013; Suri, Bargas, & Arbib, 2001). In this context, the dorsal striatum is considered to implement responses triggered by specific states or goals under the control of the critic. Chronic exposure to addictive drugs has been proposed to ‘hijack’ the functional orchestration of this relationship (Takahashi, Schoenbaum, & Niv, 2008), resulting in the development of maladaptive drug‐seeking habits (Belin, Belin‐Rauscent, Murray, & Everitt, 2013; Everitt & Robbins, 2005).

Acute, experimenter‐delivered exposure to, or short‐term history of self‐administration of addictive drugs results in increased dopamine concentration, primarily in the Acb (Di Chiara & Imperato, 1988). This increase in dopamine has been suggested to be the neurochemical basis of the reinforcing and motivational properties of drugs (Wise, 2008). The increase in Acb dopamine concentration following drug delivery has been suggested to contribute to the aberrant engagement of incentive learning mechanisms that ascribe excessive incentive value to drug‐paired cues, so‐called incentive sensitisation (Robinson & Berridge, 1993).

**Figure 1:**
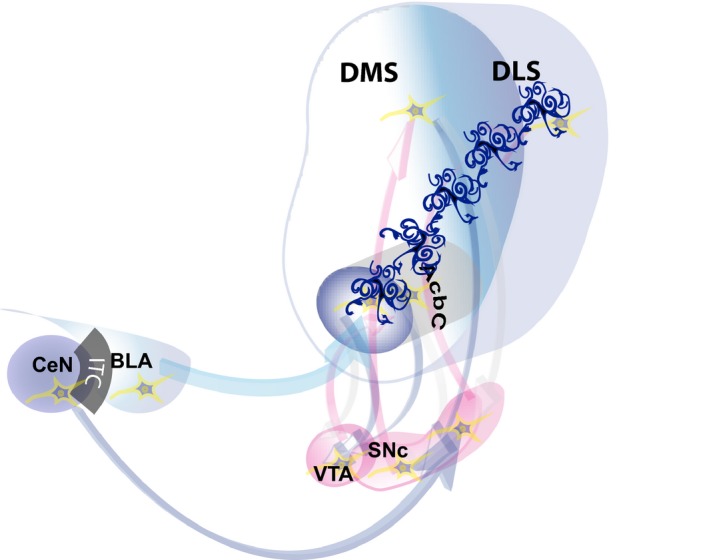
Neural systems model of the intrastriatal transitions that support the development of maladaptive compulsive drug‐seeking habits. The acquisition of cue‐controlled drug seeking depends on the interaction between the BLA and the AcbC as well as dopamine‐dependent mechanisms in the pDMS. However, when cue‐controlled drug seeking becomes habitual, its neural locus of control devolves to anterior DLS dopamine‐dependent mechanisms, the recruitment of which depends on the AcbC and the striato‐nigro‐striatal (dopamine‐dependent) ascending spiralling circuitry (involving the SNc). This functional recruitment of aDLS dopamine‐dependent control over behaviour is triggered by the BLA, but is eventually maintained by the CeN. We hypothesise that astrocytes (depicted in dark blue) may facilitate the drug‐induced intrastriatal functional transitions by bridging converging dopamine‐ and glutamate‐dependent mechanisms across several domains of the striatum. AcbC, core of the nucleus accumbens; BLA, basolateral amygdala; CeN, central nucleus of amygdala; VTA, ventral tegmental area; SNc, substantia nigra pars compacta; DLS, dorsolateral striatum; DMS, dorsomedial striatum. [Colour figure can be viewed at http://wileyonlinelibrary.com]

Critically, chronic exposure to cocaine, heroin or alcohol triggers within‐ and between‐systems adaptations that encompass the amygdalo‐striatal networks and result in the progressive functional recruitment of dorsolateral striatum (DLS) dopamine‐dependent control over behaviour (Belin & Everitt, 2008; Belin, Jonkman, Dickinson, Robbins, & Everitt, 2009; Everitt & Robbins, 2005; Volkow et al., 2006) (Figure 1). Thus, exposure to drugs, such as cocaine, shifts the balance of associative encoding from the ventral to the DLS (Takahashi et al., 2008). Similarly, in humans, non‐human primates and rats, drug‐seeking behaviour is initially dependent on, and associated with, alterations of dopaminergic mechanisms in the ventral striatum but progressively becomes dependent on dopaminergic mechanisms in the DLS (Corbit, Nie, & Janak, 2012; Cox et al., 2009; Letchworth, Nader, Smith, Friedman, & Porrino, 2001; Porrino, 2004; Volkow et al., 2006; Vollstadt‐Klein et al., 2010; Zilverstand, Huang, Alia‐Klein, & Goldstein, 2018).

The progressive transition from ventral to dorsolateral striatal dopamine‐dependent control over behaviour has been shown to be a gateway for the development of compulsive drug seeking (Giuliano, Belin, & Everitt, 2019) and, at the neural systems level, to be reliant on dopamine‐dependent functional connectivity between the AcbC and the DLS (Belin & Everitt, 2008).

This shift is dependent on the basolateral amygdala which indirectly influences the anterior DLS (aDLS) via antecedent glutamatergic mechanisms in the AcbC (Murray et al., 2015). The involvement of such glutamatergic mechanisms suggests that corticostriatal synapses may, alongside dopaminergic mechanisms, play a key role in these intrastriatal functional transitions (Figure 1).

At the level of the corticostriatal synapse, repeated exposure to various drugs of abuse has been shown to disrupt glutamate homeostasis, initially in the AcbC and subsequently in the aDLS (Ducret et al., 2015). Alterations in glutamate homeostasis have also been repeatedly shown at the prelimbic (PL) cortex → AcbC synapses (Kalivas, 2009), which function has been suggested to support behavioural adaptations to changes in the environment and associated learning processes (Barnes, Kubota, Hu, Jin, & Graybiel, 2005; Kelley, 2004). Alterations at these synapses have been suggested to contribute to the aberrant synaptic plasticity observed in animals displaying addiction‐like behaviour for cocaine (Kasanetz et al., 2010).

Importantly, astrocytes, which chemogenetic activation in the AcbC reduces the motivation of rats to self‐administer alcohol after abstinence (Bull et al., 2014), thereby revealing their role on motivational processes hitherto ascribed to dopamine‐dependent mechanisms in AcbC postsynaptic neurons, play a key role in maintaining the physiology of these corticostriatal synapses.

Astrocytes are a subpopulation of glial cells whose function has long been considered to be limited to a “basic” supportive role towards central nervous system (CNS) homeostasis (Kimelberg & Nedergaard, 2010). Indeed, astrocytes contribute to the control of cerebral blood flow (Zonta et al., 2003), extracellular pH (Kimelberg, Biddlecome, & Bourke, 1979), potassium buffering (Dietzel, Heinemann, Hofmeier, & Lux, 1980; Lothman & Somjen, 1975) and the exchange of gases through facilitation of water transport (Nielsen et al., 1997). Astrocytes have received particular attention for their pivotal role as a metabolic bridge between neurons and vascular glucose, whereby they provide neurons with the energy “fuel” they need to sustain their energy‐demanding activity (Allaman, Belanger, & Magistretti, 2011; Magistretti & Allaman, 2015).

However, over the last decade, a wealth of evidence has challenged this restrictive view of the function of astrocytes and supports a much broader and complex role for these cells in the CNS. Thus, astrocytes are increasingly considered key players in the regulation of synaptic activity and plasticity (Chung, Allen, & Eroglu, 2015; Haydon & Nedergaard, 2014; Singh & Abraham, 2017), and associated behavioural and psychological functions (Oliveira, Sardinha, Guerra‐Gomes, Araque, & Sousa, 2015). Consequently, astrocytes have been associated with the pathophysiology of several neuropsychiatric conditions including bipolar depression (Bowley, Drevets, Ongur, & Price, 2002; Cotter, Mackay, Landau, Kerwin, & Everall, 2001; Ongur, Drevets, & Price, 1998; Quesseveur et al., 2013; Sun, Liu, Yuan, Li, & Chen, 2012), schizophrenia (Feresten, Barakauskas, Ypsilanti, Barr, & Beasley, 2013; Toro, Hallak, Dunham, & Deakin, 2006) and drug addiction (Adermark & Bowers, 2016; Bull et al., 2014; Miguel‐Hidalgo, 2009; Scofield & Kalivas, 2014; Scofield et al., 2015, 2016). The supply of lactate provided to neurons by neighbouring astrocytes, long considered as merely a basic energetic supply, has recently been shown to modulate synaptic activity and to directly contribute to cocaine‐associated appetitive Pavlovian mechanisms (Boury‐Jamot et al., 2016a; Boury‐Jamot, Halfon, Magistretti, & Boutrel, 2016b).

Thus, astrocytes contribute to the influence of the incentive motivational properties of drugs on behaviour mediated by the ventral striatum (Bull et al., 2014; Scofield & Kalivas, 2014) and the aberrant learning dependent on corticostriatal circuits that contribute to the maintenance of drug addiction (Belin et al., 2009, 2013; Everitt & Robbins, 2005).

As further described below, several reviews have covered the crucial importance of astrocytic mechanisms in the ventral striatum in the context of drug self‐administration (reinforcement) and reinstatement (Kalivas, 2009; Linker, Cross, & Leslie, 2018; Scofield, 2018; Scofield & Kalivas, 2014) which have been elucidated primarily at the PL → AcbC synapse. The hypothesis developed subsequently in this review builds on these mechanisms identified at the level of the corticostriatal synapse to suggest that astrocytes may contribute to the intrastriatal functional shifts that underline the development of addiction.

Thus, at the level of the synapse, upon an action potential reaching the presynaptic terminal, a sudden increase in calcium concentration triggers the release of glutamate into the synaptic cleft wherein glutamate binds to its postsynaptic ionotropic or metabotropic (mGluR1/5/6) receptors, thereby triggering depolarisation of the postsynaptic neuron and regulation of the function of that glutamatergic synapse respectively (Niswender & Conn, 2010; Traynelis et al., 2010).

Extrasynaptic glutamate can also bind to presynaptic autoreceptors (mGluR2/3/4/7/8), the activation of which suppresses glutamate release by presynaptic terminals, thereby contributing to downregulate glutamatergic transmission (Niciu, Kelmendi, & Sanacora, 2012). Due to the highly excitotoxic nature of glutamate, its extracellular levels are tightly controlled and its clearance from the synaptic cleft is regulated via astrocytic transporters, namely GLT‐1 (Glutamate Transporter) and GLAST (Glutamate Aspartate Transporter) (Perego et al., 2000). In the intracellular compartment of astrocytes, the enzyme glutamine synthetase converts glutamate into glutamine which is subsequently released in the extracellular space, making it available to neurons as a precursor for the synthesis of glutamate within presynaptic terminals (Rose, Verkhratsky, & Parpura, 2013).

In addition to terminating glutamatergic transmission, astrocytes also control basal levels of the neurotransmitter, which, under physiological conditions, are predominantly governed by release of glutamate by the astrocytic cystine‐glutamate exchanger (xCT) (Baker, Xi, Shen, Swanson, & Kalivas, 2002). xCT couples the uptake of one molecule of cystine with the release of one molecule of glutamate at the membrane of astrocytes (Madayag et al., 2007).

Repeated exposure to various drugs of abuse, such as cocaine and nicotine has been shown to trigger a disruption of xCT function (Baker et al., 2003). As a consequence, basal extracellular glutamate levels are decreased, resulting in a decreased glutamatergic tone on presynaptic mGluR2/3 and ultimately disinhibition of glutamate release by presynaptic terminals. Thus, disruption of the astrocytic xCT activity results in both a decrease in basal glutamate levels and a facilitated glutamatergic release by presynaptic terminals, thereby altering the physiology of the corticostriatal glutamatergic synapse (Moran, McFarland, Melendez, Kalivas, & Seamans, 2005). This alteration is exacerbated by a decrease in astrocytic buffering of extrasynaptic glutamate via drug‐induced downregulation of GLT‐1 protein levels (Knackstedt, Melendez, & Kalivas, 2010; Knackstedt et al., 2009).

The functional significance of these physiological alterations at the PL → AcbC synapse has been elucidated with behavioural procedures in rodents, namely extinction/reinstatement, aiming to operationalise relapse, one behavioural hallmark of drug addiction (O'Brien, 1997). Thus, the propensity of those suffering from an addiction to relapse, even after prolonged periods of abstinence, has been suggested to be operationalised by the reinstatement of an extinguished instrumental response for the drug, a procedure initially developed by De Wit and colleagues (De Wit & Stewart, 1981, 1983).

In these procedures, rats are initially trained to self‐administer a drug for a short period of time (generally 12 days) under continuous reinforcement and are subsequently subjected to several consecutive daily instrumental extinction sessions (often 12 days). Instrumental responding is then reinstated either by an injection of the drug (drug‐induced reinstatement) (Mahler et al., 2014; Shen, Gipson, Huits, & Kalivas, 2014), a conditioned stimulus (CS, e.g. a stimulus previously presented contingently with the delivery of the drug) (cue‐induced reinstatement) (Cannella et al., 2013; LaLumiere, Smith, & Kalivas, 2012), stress (stress‐induced reinstatement) (Shalev, Erb, & Shaham, 2010) or by the context in which drug self‐administration occurred (context‐induced reinstatement) (Bossert, Marchant, Calu, & Shaham, 2013). The neural basis of reinstatement of instrumental responding for cocaine and heroin involves the mesolimbic dopaminergic system as well as a broad neural network that includes the basolateral amygdala (BLA), AcbC, prefrontal cortex (PFC) and particularly the PL → AcbC glutamatergic projections (Kalivas, 2009; Kalivas & McFarland, 2003; Knackstedt & Kalivas, 2009; LaLumiere & Kalivas, 2008; Shalev, Grimm, & Shaham, 2002). Thus, the propensity to reinstate instrumental responding for cocaine or heroin following extinction is associated with downregulation of xCT and GLT‐1 at the PL → AcbC synapse and the associated disruption of presynaptic mGluR2/3 and postsynaptic mGluR5 receptor function (Knackstedt & Kalivas, 2009; Knackstedt et al., 2009; Moran et al., 2005; Moussawi et al., 2009).

Given the putative importance of these perturbations, approaches aiming to restore astrocytic control of glutamatergic homeostasis at the PL → AcbC synapse have been investigated as potential strategies to prevent relapse.

## 2 TARGETING ASTROCYTES TO RESTORE GLUTAMATE HOMEOSTASIS: A VALID RELAPSE PREVENTION STRATEGY?

Many drugs targeting the mechanisms of astrocyte‐mediated glutamate homeostasis, such as the cephalosporin antibiotic ceftriaxone (Knackstedt et al., 2010; Trantham‐Davidson, LaLumiere, Reissner, Kalivas, & Knackstedt, 2012) or N‐acetylcysteine (NAC), have been shown to decrease instrumental responding in PL → AcbC‐dependent extinction/reinstatement procedures, as previously reviewed (Kalivas, 2009).

NAC, a cysteine prodrug which acts as a substrate for astrocytic xCT, restores basal glutamate levels in the AcbC and has shown some promise as potential treatment for addictive disorders. NAC administration has been shown to prevent drug‐ and cue‐induced reinstatement and to decrease levels of responding during extinction in rodents with a history of cocaine or heroin self‐administration (Kalivas, 2009).

The effects of NAC have been suggested to be dependent on the restoration of presynaptic mGluR2/3 function. Indeed, administration of a mGluR2/3 agonist alone diminishes reinstatement of instrumental responding for both heroin and cocaine (Bossert, Gray, Lu, & Shaham, 2006; Peters & Kalivas, 2006). Furthermore, the administration of a mGluR2/3 antagonist prevents the ability of NAC to inhibit cocaine‐induced reinstatement (Moussawi et al., 2009, 2011). NAC not only triggers an elevation of the basal glutamate levels within the synaptic cleft but it also restores the downregulated levels of GLT‐1. It is the restoration of the levels of GLT‐1 that contributes to the remediation of presynaptic mGluR2/3 tone brought about by NAC (Knackstedt et al., 2010). NAC also contributes to the remediation of postsynaptic mGluR5 function (Moussawi et al., 2009), thereby influencing both pre‐ and postsynaptic metabotropic glutamate receptors.

Importantly, treatment with NAC does not influence basal glutamate levels in drug naïve animals, suggesting that NAC specifically targets synapses wherein astrocyte‐dependent glutamatergic homeostasis has been altered by exposure to addictive drugs.

NAC, used for the treatment of paracetamol overdose (Green, Heard, Reynolds, & Albert, 2013) and as a mucolytic therapy for respiratory conditions (Sadowska, 2012), is safe and well tolerated in humans, and is therefore an excellent candidate for repurposing. Significantly, the preclinical studies discussed herein indicate NAC may be an efficacious therapeutic strategy for addictive disorders. Interestingly, a single dose of NAC has been shown to yield similar effects on glutamate homeostasis in humans as those identified in preclinical studies: cocaine‐induced disrupted glutamate levels were normalised by NAC (Schmaal, Veltman, Nederveen, van den Brink, & Goudriaan, 2012), although basal glutamate levels in this study were measured in the dorsal anterior cingulate cortex rather than in the striatum, which has been the focus of preclinical research. Importantly, treatment with NAC did not alter basal glutamate levels in healthy subjects, in line with the rodent literature.

From a therapeutic standpoint, initial clinical studies reported promising outcomes, suggesting that NAC decreased self‐reported craving and relapse following abstinence in humans who suffered from an addiction (for review see Deepmala et al., 2015). However, the subsequent and better controlled clinical trials, e.g. double blind versus placebo, have yielded outcomes that mitigated the initial enthusiasm.

For instance, when the clinical outcome is self‐reported craving and promotion of abstinence (or decreased vulnerability to relapse), NAC was shown to be mostly ineffective, except in a subset of individuals who had volitionally initiated abstinence at the onset of the trial (LaRowe et al., 2013). This important observation highlights the relevance of a dissociation to be made between the permeation by treatment of the psychological and associated neural mechanisms underlying forced‐ versus self‐imposed abstinence. NAC may indeed promote abstinence, but seemingly only in individuals who have deliberately decided to abstain, not in ongoing users.

This hypothesis is of paramount importance with regard to the phenomenology of human addiction and the relatively poor heuristic value of extinction‐reinstatement procedures on which preclinical studies have, until recently, been based (Belin‐Rauscent, Fouyssac, Bonci, & Belin, 2015).

Indeed, preclinical studies that offered evidence for the therapeutic benefits of NAC treatment tended to instantiate forced‐abstinence through instrumental extinction training. This method arguably does not capture the self‐initiated nature of abstinence in addicted individuals who stop taking drugs in response to mounting negative consequences (Peck & Ranaldi, 2014).

**Figure 2:**
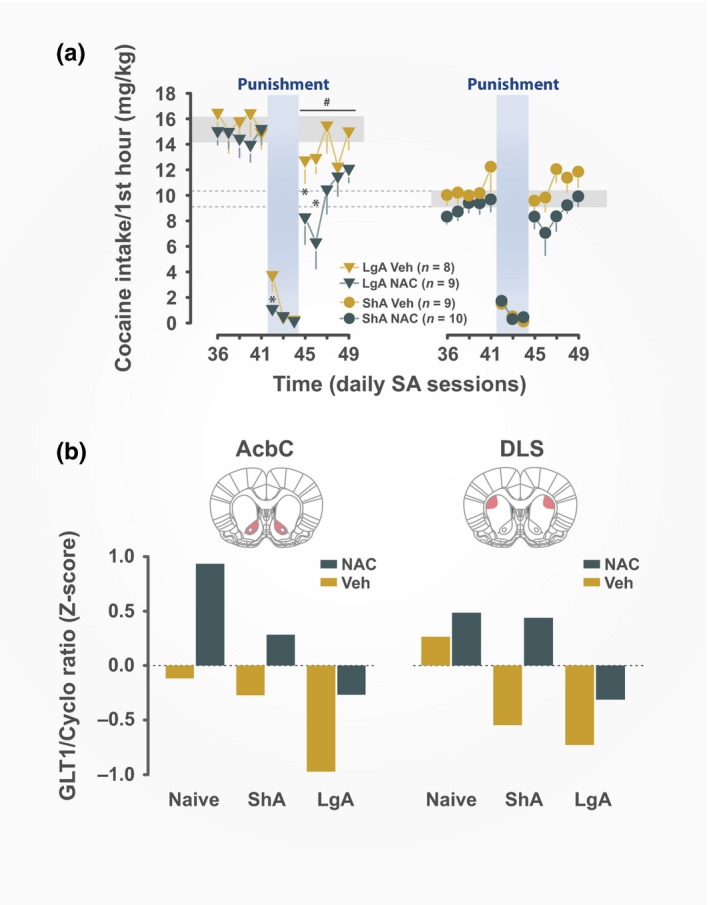
NAC promotes self‐maintained abstinence in the face of adverse consequences and rescues cocaine‐induced decreases in GLT‐1 levels in the ventral and dorsolateral striatum. (a) Punishment of cocaine self‐administration triggers a drop in responding both in rats with an history of short‐ or extended access to the drug (ShA and LgA respectively). Upon cessation of punishment, NAC‐treated LgA rats did not fully resume their pre‐punishment level of cocaine self‐administration and no longer displayed escalation, in contrast with vehicle‐treated LgA rats. (b) NAC rescued cocaine‐ and access‐dependent decreases in GLT‐1 protein levels both in the AcbC and DLS (adapted from Ducret et al., 2015, originally published under a Creative Commons Attribution License (CC BY)). Cyclo, cyclophilin; AcbC, core of the nucleus accumbens; DLS, dorsolateral striatum; NAC, N‐acetylcysteine; ShA, short access; LgA, long access; Veh, vehicle; SA, self‐administration. [Colour figure can be viewed at http://wileyonlinelibrary.com]

Back translating these observations from bedside to bench, Ducret et al. (2015) tested the hypothesis that in rats with a history of escalated cocaine self‐administration, NAC may facilitate self‐initiated abstinence in the face of negative consequences and help restore control over drug intake (Ducret et al., 2015). In that study, rats were trained to self‐administer cocaine under extended (6‐hr) (LgA) or short (1‐hr) (ShA) access. Extended access to cocaine has been shown to trigger a rapid increase in drug intake, also known as escalation (Ahmed & Koob, 1999, 2005). Rats were subsequently exposed to punishment of the instrumental response by contingent presentations of electric foot shocks. Chronic NAC treatment promoted self‐abstinence in the face of negative consequences in LgA rats as measured by a facilitated decrease in instrumental responding in the presence of punishment, as compared to vehicle‐treated rats. The effect of NAC persisted when negative consequences were no longer present, thereby revealing the ability of NAC to restore control over drug intake at relapse in LgA rats (Figure 2a).

These data provide causal evidence that NAC promotes abstinence and restores control over intake in individuals who volitionally self‐abstain because of negative consequences. At the neural systems level, NAC rescued the cocaine‐induced downregulation of the astrocytic GLT‐1 protein levels observed in LgA rats, not only in the AcbC, as had been previously described, but also in the aDLS, in which the drug‐induced downregulation of GLT‐1 had not yet been described (Figure 2b).

This observation is consistent with the wealth of evidence for a progressive devolvement of control over behaviour to aDLS dopamine‐dependent mechanism over the course of drug exposure in humans, non‐human primates and rodents (Belin & Everitt, 2008; Ersche et al., 2012a, 2012b; Jonkman, Pelloux, & Everitt, 2012).

Considering the role of the aDLS in mediating drug‐seeking habits (Belin‐Rauscent, Everitt, & Belin, 2012; Corbit et al., 2012) and compulsive drug seeking (Giuliano et al., 2019; Jonkman et al., 2012), these data suggest that astrocyte‐dependent alterations in the homeostasis of corticostriatal glutamatergic synapses span the different corticostriatal functional loops (Haber, 2016), far beyond the PL → AcbC synapse. Indeed, the PL also projects to the posterior dorsomedial striatum (pDMS) (Vertes, 2004), which is involved in mediating instrumental responses underlined by action‐outcome (A‐O) associations and early established cue‐controlled cocaine seeking behaviour (Murray, Belin, & Everitt, 2012a). In contrast, aDLS‐dependent habits are controlled by another territory of the mPFC, namely the infralimbic cortex (IL), which projects to the AcbS and the central amygdala (CeN) (Vertes, 2004), another key component of the habit system (Belin et al., 2013; Lingawi & Balleine, 2012; Murray et al., 2015).

**Figure 3:**
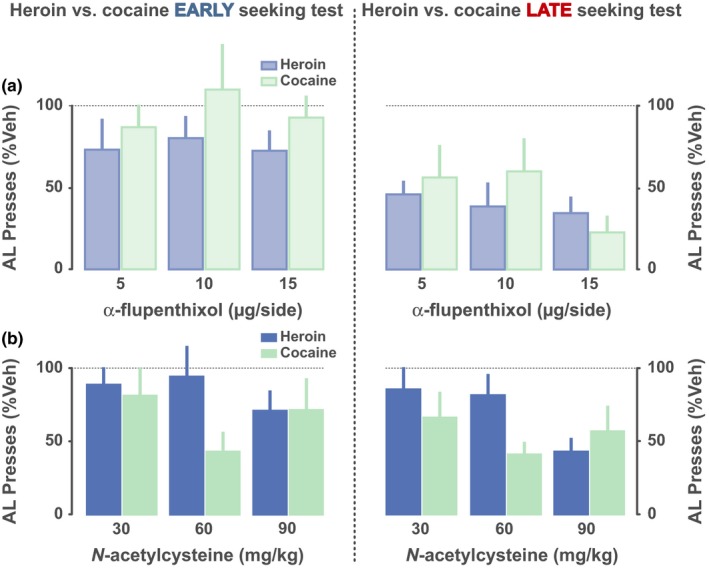
NAC reduces aDLS dopamine‐dependent drug‐seeking habits. (a) Overtraining under a second order schedule of reinforcement for cocaine or heroin promotes the devolvement of control over behaviour to aDLS dopamine‐dependent mechanisms in that only late, and not early, stage drug‐seeking behaviour is dose‐dependently decreased by bilateral intra‐aDLS infusions of the dopamine receptor antagonist α‐flupenthixol. (b) N‐acetylcysteine is equally effective at reducing cocaine and heroin seeking behaviour at early, aDLS‐independent, and late, aDLS dopamine‐dependent, performance stage (Hodebourg et al., 2018, originally published under a Creative Commons Attribution License (CC BY)). AL, active lever; Veh, vehicle; aDLS, anterior dorsolateral striatum. [Colour figure can be viewed at http://wileyonlinelibrary.com]

Recent evidence showed that NAC markedly decreases cue‐controlled cocaine and heroin seeking behaviour in rats trained under a second order schedule of reinforcement. These effects were observed both at early and late stages of training, when the behaviour is controlled by aDLS‐independent and aDLS‐dependent mechanisms respectively (Hodebourg et al., 2018; Murray, Everitt, & Belin, 2012b) (Figure 3).

Thus, in rats trained to seek cocaine or heroin for protracted periods of time under the control of contingent presentations of drug‐paired CSs, acting as conditioned reinforcers, drug seeking is initially impervious to manipulations of glutamate or dopamine receptors in the aDLS, and is instead decreased by lesions, inhibition of, or dopamine receptor blockade in, the network involving the BLA, AcbC, posterior DMS (pDMS) and orbitofrontal cortex (Belin & Everitt, 2008; Hutcheson & Everitt, 2003; Murray et al., 2012a, 2015; Vanderschuren, Di Ciano, & Everitt, 2005). In marked contrast, when cue‐controlled drug‐seeking behaviour is well established, it becomes reliant on aDLS dopamine‐dependent mechanisms and their control by the CeN as they are dramatically decreased by aDLS dopamine receptor blockade, inactivation of the CeN or functional disconnection between the two (Murray et al., 2015) (Figure 1).

The effect of NAC on DLS‐dependent drug‐seeking behaviour is in agreement with the observation by Corbit and colleagues that NAC restores A‐O control over behaviour in rats whose instrumental response was habitual and dependent on the aDLS (Corbit, Chieng, & Balleine, 2014).

Together with the evidence that cocaine seeking habits are also mediated by glutamatergic mechanisms in the aDLS (Vanderschuren et al., 2005), these observations suggest that astrocyte‐dependent glutamatergic mechanisms interact with dopaminergic mechanisms in different loops of the corticostriatal circuitry to regulate the balance between goal‐directed and habitual control over behaviour.

This is far reaching, considering the large anatomical and functional territories one astrocyte is able to regulate: a single astrocyte can contact up to 140,000 synapses in a rat brain (Bushong, Martone, Jones, & Ellisman, 2002) and up to 2 million synapses in the human brain (Bushong et al., 2002; Oberheim et al., 2009). Within the cytoarchitectonic context of the striatum discussed before, this would suggest that a single astrocyte can define a functional unit and facilitate integration of synaptic mechanisms across several functional territories of the striatum. The unique structural and functional properties of astrocytes within the striatum place these cells in an excellent position to contribute to the intrastriatal functional coupling between, and shifts in the locus of control from ventral to dorsal territories (Belin & Everitt, 2008) that support the development of drug addiction.

## 3 ASTROCYTES, THE GREAT ORCHESTRATORS OF DRUG‐ASSOCIATED STRIATAL ALTERATIONS

Astrocytes are not only involved in maintaining glutamate homeostasis and regulating glutamatergic transmission, but they also express a broad range of neurotransmitter receptors at their membrane depending on their cellular (neuronal) environment, including dopamine (Khan, Koulen, Rubinstein, Grandy, & Goldman‐Rakic, 2001), endocannabinoids (Navarrete & Araque, 2010), GABA (Lee, McGeer, & McGeer, 2011) and noradrenaline (Ding et al., 2013; Lerea & McCarthy, 1989) receptors. The activation of these receptors triggers direct modulation of intracellular calcium levels (for a detailed review see Moraga‐Amaro, Jerez‐Baraona, Simon, & Stehberg, 2014) either restricted locally or, if large enough, affecting more distant astrocytes through calcium waves (Charles, Merrill, Dirksen, & Sanderson, 1991; Cornell‐Bell, Finkbeiner, Cooper, & Smith, 1990). Indeed, calcium waves can spread from one astrocyte to another throughout the syncytium that relies on gap junctions (Nagy & Rash, 2000).

Modulation of intracellular calcium levels in astrocytes can eventually trigger the release of neurotrophic factors, known as gliotransmitters, such as ATP, D‐serine, TNF‐alpha or, as previously discussed, glutamate, all of which play an important role in the regulation of synaptic activity (Beattie et al., 2002; Cotrina, Lin, & Nedergaard, 1998; Henneberger, Papouin, Oliet, & Rusakov, 2010). Thus, the release of glutamate by astrocytes is far from restricted to the aforementioned homeostatic mechanism brought about by xCT. The ability of astrocytes to release glutamate within the synaptic cleft is highly dynamic and involves various pathways including calcium‐dependent exocytosis, reverse operation of glutamate transporters or release through purinergic anion channels, volume‐regulated anion channels and even connexons (for review see Malarkey & Parpura, 2008).

**Figure 4:**
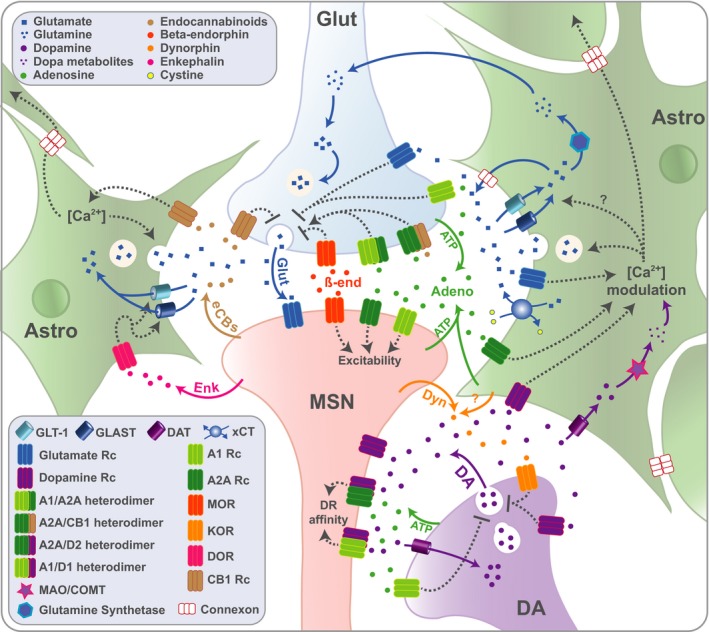
Between systems‐mediated glutamate and dopamine signalling within the quadripartite synaptic microenvironment. Glutamate is released into the synaptic cleft where it is able to bind to its postsynaptic, presynaptic and astrocytic receptors. Glutamate reuptake is governed by astrocytic transporters (GLT‐1 and GLAST) and can be either enzymatically degraded into glutamine or released into the synaptic cleft by: exocytosis, the exchanger xCT, the connexons or reversal of the transporters. Dopamine (DA) is released by dopaminergic neurons and binds to D1‐ and D2‐like postsynaptic and astrocytic receptors (for the sake of clarity the two receptors are displayed here on the same MSN; however, such co‐localisation is scarce in the striatum, observed only in up to 5% or 20% of the MSNs in the dorsal striatum and nucleus accumbens respectively). DA is quickly taken up back into the presynaptic terminal by the dopamine transporter (DAT). However, DAT is also expressed on striatal astrocytes in which DA can be degraded by enzymatic reactions leading to formation of reactive oxygen species able to influence intra‐astrocytic calcium levels. Adenosine is released by astrocytes or is transformed by extracellular enzymatic reactions from neuronal ATP. A1 receptors are expressed presynaptically both on the dopaminergic and glutamatergic terminals where their activation inhibits neurotransmitter release. A1 and A2A receptors are expressed postsynaptically and modulate the neuronal excitability of MSNs. Adenosinergic receptors also have the ability to form heterodimers: on glutamatergic projections, activation of the heterodimers A1/A2A and A2A/CB1 modulates glutamate release (depending on the extracellular level of adenosine) and dimerisation of postsynaptic A2A with D2, or A1 with D1, modulates negatively the affinity of dopaminergic receptors to dopamine. Activation of astrocytic A2A receptors enhances glutamate release from astrocytes, potentially by increasing calcium levels. Postsynaptic endocannabinoids bind to CB1 receptors expressed both on presynaptic glutamatergic projections where they inhibit neurotransmitter release and on astrocytes where they increase glutamate release. Dynorphin, released by postsynaptic neurons (and potentially by astrocytes), binds to κ‐opioid receptors expressed on dopaminergic terminals and their activation inhibits dopamine release. Postsynaptic Enkephalin binds to δ‐opioid receptors which activation in astrocytes has been shown to upregulate the expression of astrocytic glutamate transporters. β‐endorphin binds both pre‐ and postsynaptically on μ‐opioid receptors. Their activation on glutamatergic projections inhibits glutamate release while postsynaptically, it modulates negatively the neuronal excitability of MSNs. Astro, astrocyte; Glut, glutamate; DA, dopamine; ATP, adenosine triphosphate; Adeno, adenosine; eCBs, endocannabinoids; MSN, medium spiny neurons; DR, dopamine receptors; Dyn, dynorphin; D1, dopamine receptor D1; D2, dopamine receptor D2, Enk, enkephalin; B‐end, beta‐endorphin; A1, adenosine 1 receptor; A2A, adenosine 2a receptor; MOR, μ‐opioid receptor; KOR, κ‐opioid receptor; DOR, δ‐opioid receptor; CB1, cannabinoid receptor 1; GLT‐1, glutamate transporter; GLAST, glutamate aspartate transporter; xCT, cystine‐glutamate exchanger; Ca^2+^, calcium. [Colour figure can be viewed at http://wileyonlinelibrary.com]

Even though the mechanisms of glutamate release by astrocytes have been identified, the conditions under which astrocytes employ one pathway rather than another remains to be elucidated. Further research is warranted to better understand whether these mechanisms operate concurrently or independently, and if the same pathways are recruited under physiological and pathological conditions such as drug addiction. Nevertheless, it is important to highlight that direct communication between neurons and astrocytes also control extracellular levels of glutamate. Stimulation of adenosine receptors (A2A Rcs) expressed on astrocytes by adenosine released by neurons triggers astrocytic glutamate release (Li, Nomura, Aihara, & Nishizaki, 2001; Nishizaki et al., 2002; for a detailed review see Boison, Chen, & Fredholm, 2010). Astrocytes also influence glutamate homeostasis via delta opioid receptors. The activation of these G‐coupled protein receptors result in the upregulation of excitatory amino acid transporter protein levels in astrocytes (Liang et al., 2014), thereby representing a potential converging mechanism between endogenous opiates and astrocyte‐mediated regulation of synaptic plasticity within the striatum. Finally, cannabinoid‐1 receptors, which are densely expressed in the striatum following a medial‐dorsal gradient (Martín et al., 2008), are also present at the membrane of astrocytes. Activation of these receptors via neuronal endocannabinoids generates astrocytic glutamate release, which has recently been shown to play a role in the homotypic potentiation of medium spiny neurons in the striatum (Martin, Bajo‐Graneras, Moratalla, Perea, & Araque, 2015), a mechanism that is potentially involved in the functional synchronisation of adjacent striatal territories (Figure 4).

The pivotal role of astrocytes in glutamate homeostasis has been relatively well described. However, the contribution of these cells to dopamine homeostasis and its impact on glutamatergic transmission remains poorly understood, despite increasing evidence from both *in vitro* and *in vivo* studies that astrocytes contribute to the function of dopaminergic synapses (Jennings & Rusakov, 2016). Thus, under physiological conditions, following release into the synaptic cleft by the presynaptic neuron, dopamine binds to D1‐ or D2‐like postsynaptic receptors, or to its D2‐like presynaptic receptors. The termination of dopamine transmission is governed by two mechanisms: a specific reuptake of dopamine via the dopamine transporter (DAT) and enzymatic catabolism of dopamine by the monoamine oxydases (MAO) and the cathecol‐o‐methyltransferase (COMT). Importantly, the DAT is not only expressed on the membrane of presynaptic terminals of dopamine neurons, it is also expressed on astrocytes (Inazu et al., 1999; Karakaya, Kipp, & Beyer, 2007), which, alongside the former, also express the MAO and COMT (Hitri, Hurd, Wyatt, & Deutsch, 1994) (Hansson & Sellstrom, 1983; Huang, Dragan, Freeman, & Wilson, 2005) (Figure 4).

Apart from their role in dopamine clearance, cultured astrocytes have been shown to respond to dopamine, in that direct application of dopamine modulates cytosolic calcium signalling in astrocytes. These effects occur both in a receptor dependent manner, through D1/D2 receptors (Jennings et al., 2017) and in a receptor‐independent manner, whereby the reactive oxygen species generated by the cytosolic degradation of dopamine by MAO directly control calcium signalling (Vaarmann, Gandhi, & Abramov, 2010).

Collectively, these data suggest an important role of the DAT, the gateway for dopamine into astrocytes, in the coupling between dopaminergic synaptic activity and calcium signalling.

Thus, investigation of the role of astrocytes in the regulation of striatal synaptic physiology should go beyond the prototypic tripartite glutamatergic synapse and should consider the striatal dopaminergic synapse in a quadripartite synaptic environment. A more complete understanding of this quadripartite system is paramount for our understanding of drug addiction. The contribution of dopamine signalling to the homeostasis and function of the striatal quadripartite synaptic microenvironment remains to be elucidated but could represent the cellular mechanism by which drugs of abuse, which aberrantly increase dopamine levels in the striatum, trigger long‐lasting alterations of glutamate homeostasis that span the entire striatum.

Furthermore, endogenous systems, namely endocannabinoids, endogenous opiates and the adenosinergic system, have been shown to influence glutamatergic and dopaminergic homeostasis as well as the dynamic function of astrocytes in the striatum (for review see Fouyssac, Everitt, & Belin, 2017) (Figure 4). The cross‐talk between these systems and astrocytes is at the crossroads of drug reinforcement in the ventral striatum, regulation of the control over instrumental responding by A‐O and S‐R associations in the dorsal territories of the striatum, and ultimately striatal synaptic plasticity mechanisms which are hijacked by drugs of abuse. Indeed, repeated exposure to cocaine has been associated with a metaplasticity phenomenon within the AcbC, i.e. a deficit in the ability to develop long‐term potentiation (LTP) or depression (LTD) (Moussawi et al., 2009), and with an anaplasticity phenomenon, i.e. a permanent impaired LTD (Kasanetz et al., 2010), both mechanisms dependent on glutamate homeostasis.

## 4 CONCLUSION

The purpose of this review was to summarise experimental evidence in support of the involvement of astrocytes in the dysregulation of the glutamate homeostasis induced by exposure to drugs of abuse across the functional domains of the striatum and offer a broader mechanistic view of the striatal quadripartite synaptic microenvironment. The disruption of the homeostasis and function of this striatal microenvironment may contribute to the emergence of maladaptive drug‐seeking habits and ultimately compulsive behaviour, the hallmark of addiction. A better understanding of the neural, behavioural and psychological consequences of the restoration of glutamate levels by NAC both in preclinical and clinical models has helped shed a new light on the cellular mechanisms in the striatum that contribute to several facets of addiction. However, the drug‐induced alteration of glutamate homeostasis characterised by the downregulation of astrocytic xCT and GLT‐1 may only be the tip of the iceberg of the many adaptations these cells may undergo in response to drug exposure, including those associated with dopaminergic transmission. Further research is warranted better to understand the physiology of the quadripartite synaptic microenvironment which, we hypothesise, represents the functional unit of the striatum, and the extent to which interactions between dopaminergic and glutamatergic systems are channelled by astrocytes. The molecular landscape of the quadripartite synaptic microenvironment and its associated physiology might differ between individuals and such difference may represent molecular and cellular vulnerability factors which could account for the inter‐individual propensity to develop maladaptive drug seeking habits and eventually drug addiction (Anthony, Warner, & Kessler, 1994).

## CONFLICT OF INTEREST

The authors have no financial or potential conflicts of interest to declare.

## AUTHOR CONTRIBUTIONS

MF and DB have contributed equally to the writing of the manuscript.
