# Supplementary material for: Beyond drug‐induced alteration of glutamate homeostasis, astrocytes may contribute to dopamine‐dependent intrastriatal functional shifts that underlie the development of drug addiction: A working hypothesis
Source: Eur J Neurosci. 2019 May 14;50(6):3014–27. doi: 10.1111/ejn.14416 (PMC6852203; doi:10.1111/ejn.14416)
Supplement: Supplementary file 1 [file EJN-50-3014-s001.docx]

| Beyond drug-induced alteration of glutamate homeostasis, astrocytes may contribute to dopamine-dependent  intrastriatal functional shifts that underlie the development of drug addiction. A working hypothesis. | |
| --- | --- |
|  | [Belin, David](javascript:popWindow('ejn?PARAMS=xik_5SUjnX6JCLoAN5YLbg2YBdbrxMA5jiWYSEGGJpjEBA1eUKpyCZZvALEA4MTJ1BhEPpQvgvxNyGtZ4Sn3PzMWrGDzR2Jr8CKtB9F4W8R4UAh8S5MX6vBgQvVhfqZYy8FCYipLUtg9XiaRkTPYyk6QLvhXczua1STbr6bwFNhhjJRyj7G','mailpopup_1340',%20900,%20775);); Fouyssac, Maxime |

_____________________________________________________________________________________

| Review timeline: | Submission date: 30/11/18  Editorial Decision: Minor Revision  Revision Received: 22/02/19  Editorial Decision: Minor Revision  Revision Received: 18/03/19  Accepted: 26/03/19 |  |
| --- | --- | --- |
|  |  |  |

Editor: Paul Bolam

Reviewer 1: Vinay Parikh

Reviewer 2: Dominique Massotte

_____________________________________________________________________________________

1st Editorial Decision	30/12/18

_____________________________________________________________________________________

A potential role for striatal astrocytes in the dopamine-dependent intrastriatal functional shifts that underlie the development of drug addiction 

Dear David, 

Your manuscript has been reviewed by two external reviewers as well as by the Editorial Team.  We are pleased to say that both reviewers appreciate your review but both raise a few issue that will need to be addressed in a revised version.   

Reviewer 1 raises the point about the title/focus of the review (also reviewer 2); maybe tighten up the title and define more clearly what will be discussed.  He/she suggests including something about the targetting of GLT-1 and including the description of the model in Fig 3 in the text rather than just the legend, both of which we consider will improve the manuscript. Reviewer 2 is primarily concerned about the focus/clarity and the failure to cite some literature that seems to be central to the topic of the review.  These points should be relatively easy to address.  Please carefully respond to each of the reviewers' comments in a revised version of the manuscript. 

We noted the following additional points that should be addressed in the revised version 
- Please include a conflict of interest statement and an author contribution statement 
- Please convert in-text references and the reference list to EJN style. 
- We also need a list of abbreviations. 
- Legend to Fig 1.  Clarify in A that the x axis is time in min(?).  What is FR5?  It is confusing as the abbreviation LgA is used for the 'extended time'.  B: GLT1 protein levels need units. 
- Legend to Fig 2. Probably also needs to be made more accessible to the non-specialist,  e.g. NAC used in legend and N-acetylcysteine used in figure;define aDLS. 

When revising the manuscript, please embolden or underline major changes to the text so they are easily identifiable and DO NOT leave 'track change' formatting marks in your paper.  Please ensure that you provide a text and a figure file for the Graphical Abstract (as detailed in the instructions below).  When carrying out your revisions please refer to the checklist below and visit the EJN author guidelines at www.ejneuroscience.org 

When finalized, please upload your complete revised manuscript onto the website, as a Word file (.doc, or .docx).  Please also ensure that a complete set of tables and figures is included as separate files, even if these have not changed from the originals.  At this stage it is necessary to provide high resolution figures.  Please see important instructions below. 

Please go into https://mc.manuscriptcentral.com/ejn - Author Centre - manuscripts with decisions where you will find a 'create a revision' link under 'actions'. We ask that you please indicate the way in which you have responded to the points raised by the Editors and Reviewers in a letter. Please upload this response letter as a separate Word (.doc or PDF) file using the file designation "Authors' Response to Reviewers" when uploading your manuscript files.  Please DO NOT submit your revised manuscript as a new one.  Also, please note that only the  Author who submitted the original version of the manuscript should submit a revised version. 

If you are able to respond fully to the points raised, we would be pleased to receive a revision of your paper within 12 weeks. 

Thank you for submitting your work to EJN. 

Best wishes for the New Year, 

Paul & John 
co-Editors in Chief, EJN 


Reviews: 

Reviewer: 1 

Comments to the Author 
The present review highlights major advances in understanding the potential role of astrocytes in modulating synaptic transmission in striatal microcompartments and drug seeking behaviors. The authors nicely summarized findings from a plethora of studies that illustrate aberrations in astrocytic control of glutamate homeostasis in the Acb and DLS occurring as a consequence of chronic drug use and how these mechanisms contributes to relapse. They also supported this hypothesis by presenting evidence from preclinical pharmacological studies that show NAC facilitate self-initiated abstinence and prevent relapse by restoring striatal glutamate function. Another component of the review was the interactions between the dopaminergic system and astrocytes that converge on calcium signaling. These mechanisms were discussed to explain the dopaminergic regulation of glutamate homeostasis and addictive behaviors using a striatal quadripartite synaptic model.   Overall, the review is very well-written and includes good illustrations. I have a few minor suggestions that should be considered for the revisions. 

1) The title of the review gives the impression that astrocytic regulation of the dopaminergic striatal microcircutry is the central tenet of this thesis. However, the review is predominantly focused on glutamate homeostasis which is fine given the fact that most of the evidence involving glial mechanisms of drug addiction interfaced with glutamatergic transmission. The authors were articulative in integrating dopaminergic mechanisms to the existing tripartite glutamate synapse model and how it may shape drug seeking behavior given the limited evidence, but this piece was incorporated only at the later part of the review. I suggest keeping either the title more general (e.g. something along the lines of astrocytic mechanisms of addiction) or make it more relevant to glutamate-dopamine interactions. 

2) For the section on therapeutic interventions to restore glutamate homeostasis, studies focused on normalizing xCT with NAC were only summarized. There was no discussion on strategies to optimize GLT-1 function using cephalosporin antibiotics as an approach to combat drug abuse. The authors did cite one study that did not find any differences in the GLT-1 protein in LgA rats but there are extensive evidence that most addictive drugs alter GLT-1 expression and/or glutamate uptake in the Acb and DS. Therefore, I suggest adding a subsection on pharmacological approaches that target GLT-1 as another strategy to combat craving and relapse. 

3) The legend for the hypothetical quadripartite model figure (#3) is too extensive and the text could be curtailed. It might be a good idea to restructure the last part of the section on Astrocytes to discuss the framework for this model by integrative some of the text presented in the legend. 


Reviewer: 2 

Comments to the Author 
In this review, M. Fouissac and D. Belin aim at covering evidence from the literature for a role of striatal astrocytes in the development of addiction. Functional implication of glial cells remains poorly investigated to date and, more so, in the context of drug addiction. This review is thus tackling a very important aspect by addressing the molecular mechanisms underlying the fine-tuning of dopamine signalling as well as its modulation in pathological conditions. However, the content of the manuscript appears very disappointing and the focus remains unclear because a large part of the review relates to the modulation of neuronal connectivity in various models of addiction without any clear link to the intended topic. 
Following a brief introduction raising interest in the functional role of astrocytes, the first section of the review is devoted to the modulation of neuronal connections influencing striatal dopamine level in various behavioural paradigms. Glutamate transporters present in astrocytes are superficially broached and do not bring any real insight. The second part of the review is entirely devoted to the potential therapeutic interest of N-acetylcysteine to prevent relapse and surprisingly concludes on the importance of astrocyte-dependent glutamatergic component on dopaminergic mechanisms in different models related to addiction and the high number of connections one astrocytic cell can provide. Again, the link with the functional role of astrocytes remains elusive. The last part aims at reviewing the molecular components expressed on astrocytes that may actively contribute to the control of glutamatergic homeostasis and influence dopaminergic signalling. However, this part mixes transporters and GPCRs and would benefit from a better organization of its content. Finally, the authors propose to integrate the astrocytic component in a more comprehensive model of the striatal synapse. However, this model is only presented in the legend of figure 3 and appears somewhat at odd with the content of the main text. In addition, no reference to previous literature is included for the already known molecular aspects. 
Beside the absence of a clear guideline, a major drawback of the review is the lack of references suggesting a functional role for astrocytes in drug addiction. See for example Bowers & Kalivas 2003 EJN, Green-Sadan et al 2003 EJN, Nam et al 2013 J Neurosci, Arezoomadan et al 2016 Neurochem Res, Arezoomadan et al 2016 Brain res Bull, Kitamura et al 2010 Leg Med. 
For the molecular aspects, the authors could also refer to very recent reviews by M.D. Scoffield 2018 Biol Psy on the role of astroglial GPCRs and mechanisms of glutamate release and by Linker et al 2018 EJN on glial mechanisms underlying substance abuse addiction. I would suggest reshaping the current manuscript to provide a complementary, original and focused viewpoint on the functional role of astrocytes in the striatum derived from the integrated perspective of the models and plasticity/relevance of neuroanatomical connections. 

Other points 
- P12 line 13 :To the best of my knowledge, there is still no evidence for dopamine D1-adenosine A1 heteromers in the striatum. 

- The work by Bull et al 2014 is instrumental to the content of the present review to support the role of astrocytes in drug addiction. Therefore, the experimental data supporting this conclusion should be described in more detail. 

- References Ducret et al 2015 and Hodebourg et al 2018 are incomplete 

- The message and added value of figures 1 and 2 are both unclear in the intended context of the review. 

------------------------ 
____________________________________________________________________________________

Authors’ Response

_____________________________________________________________________________________

Dear Paul and John, 


We would like to thank you and the reviewers for the positive appraisal of, and the constructive comments we received on, the original version of our manuscript. 
We have taken into all account all of the comments we have received in revising our manuscript which has been substantially amended (amendments shown in dark pink). 
Thus, we have amended the title of the manuscript in order better to capture the fact that our quadripartite synapse hypothesis builds on the understanding of the contribution of astrocytes to the drug-induced alterations of the glutamatergic homeostasis, hitherto almost exclusively studies at the prelimbic◊ accumbens core synapse. 
We have also completely reorganised the structure of the manuscript better to introduce the importance of intrastriatal functional transitions over the course of drug addiction and then introduced the potential role of astrocytes within this framework. We have added a figure that illustrates the neural circuits associated with the dopamine-dependent intrastriatal functional transitions. 
We believe most of the reviewers’ concerns are related to the different views we have with regards to what addiction is, and how it is best operationalised in rodents. While we are aware there are no ultimate models of addiction in non-human species, we have been arguing for the last 15 years that addiction stems from the loss of control over drug seeking habits only in some vulnerable individuals who therefore move from recreational drug seeking and taking to the maladaptive compulsive drug seeking behaviour that characterises addiction. 
Within this framework, we have offered the first evidence of this individual vulnerability to develop addiction-like behaviour in the rat and demonstrated that drug seeking habits, which depend on the progressive functional recruitment of dorsolateral striatum-dopamine dependent control over behaviour, the focus of this review, represent a gateway for the development of compulsivity. 
Having identified that N-acetylcysteine, which restores goal-directedness in rats whose instrumental behaviour was under habitual, S-R control (work from Balleine), decreases habitual cocaine, as well as heroin DLS-dependent seeking responses and that it promotes self-initiated abstinence in rats with an history of cocaine escalation, we sought to offer a novel hypothesis focusing on astrocytes and their potential role in the intrastriatal shifts that underlie the development of compulsive drug seeking habits, the hallmark of addiction. In doing so, we have attempted to offer as scholarly as possible an overview of our understanding of the contribution of astrocytes to drug reinforcement and extinction/reinstatement, fully aware that the procedures deployed in the associated studies fall short of promoting the intrastriatal transitions this review interests itself with. 
While briefly mentioning the limitations of these procedures we wanted to demonstrate our respect to the seminal discoveries, especially from Peter Kalivas’ lab and trainees. However, we only used the accrued knowledge, which has been extensively reviewed elsewhere, to build on a neural systems model of the contribution of striatal astrocytes to drug seeking habits and associated compulsivity. 
For these reasons, our review, which now states better that it aims to bring up a new hypothesis, may seem only superficially to discuss specific aspects of the current knowledge of the detailed molecular mechanisms whereby astrocytes contribute, in the nucleus accumbens, to reinstatement performance but we chose not to delve into each detail because these have been extensively reviewed recently and they fall beyond the scope of this work. 
We believe our manuscript has been greatly strengthened in the process and we hope it will now be considered suitable for publication in European Journal of Neuroscience. 
An itemised response to yours and the reviewers’ comments is appended below. 


With my best wishes, 

David 
  
Response to reviewers: 


Reviewer: 1 

Comments to the Author 

1) The title of the review gives the impression that astrocytic regulation of the dopaminergic striatal microcircutry is the central tenet of this thesis. However, the review is predominantly focused on glutamate homeostasis which is fine given the fact that most of the evidence involving glial mechanisms of drug addiction interfaced with glutamatergic transmission. The authors were articulative in integrating dopaminergic mechanisms to the existing tripartite glutamate synapse model and how it may shape drug seeking behavior given the limited evidence, but this piece was incorporated only at the later part of the review. I suggest keeping either the title more general (e.g. something along the lines of astrocytic mechanisms of addiction) or make it more relevant to glutamate-dopamine interactions.

Point taken. We have amended the title that now reads: 
Beyond drug-induced alteration of glutamate homeostasis, astrocytes may contribute to dopamine-dependent intrastriatal functional shifts that underlie the development of drug addiction. A working hypothesis. 

2) For the section on therapeutic interventions to restore glutamate homeostasis, studies focused on normalizing xCT with NAC were only summarized. There was no discussion on strategies to optimize GLT-1 function using cephalosporin antibiotics as an approach to combat drug abuse. 

Cephalosporin antibiotics, such as Ceftriaxone that restore glutamate homeostasis, have indeed been studied as a potential pharmacological treatment to prevent relapse in preclinical studies. We now refer to it. 

However, the procedures used in these studies are based on extinction/reinstatement tests that following a 10-12 day period of self-administration. These procedures fall short of recapitulating the human situation in that i) they do not recruit intrastriatal functional transitions, they are based on too short an exposure to drug SA ii) and iii) fail to consider the nature of protracted drug seeking in addiction, as opposed to reinforcement. Similarly, relapse in humans seldom, if ever, occurs following extinction while reinstatement represents also the acquisition of responding under the conditioned reinforcing properties of drug-paired cues having learnt previously a Response-No US association overlaid on the initial response-US association. 
Taken together these aspects of the procedure make it difficult really to understand the psychological mechanisms involved in instrumental performance measured in extinction/reinstatement tests and we do not want to delve in these considerations in the present review, nor was it our objective to offer an exhaustive review of the different strategies used to restore glutamate homeostasis in the context of these procedures. 
Similarly, alcohol intake in alcohol-preferring rats is dissociable from their propensity to develop compulsive drug-seeking habits (Giuliano et al., 2018, 2019). We do not think this review should discuss these particular issues that would detract the reader from the scope, namely astrocytes and their potential role in mediating joint, convergent dopamine/glutamate homeostasis alterations. 
Instead our goal here was to help the broad readership, who may somehow be naïve to these considerations, understand how pharmacological tools have helped identify the potential role of astrocyte-dependent mechanisms in relapse and how the transfer to the clinic did not receive the success that was initially expected, thereby warranting new preclinical investigations that have now led to the understanding that astrocyte-dependent mechanisms occur beyond the prelimbic⎝AcbC synapse and are potential good targets in the maintenance of self-initiated abstinence and in helping individual relinquish DLS-dependent drug seeking habits. 

The authors did cite one study that did not find any differences in the GLT-1 protein in LgA rats but there are extensive evidence that most addictive drugs alter GLT-1 expression and/or glutamate uptake in the Acb and DS. Therefore, I suggest adding a subsection on pharmacological approaches that target GLT-1 as another strategy to combat craving and relapse. 

We do not understand the point made by this reviewer: escalation, while very interesting, has no heuristic value whatsoever with craving (which is a subjective state that cannot be measured in animals) or relapse. As a matter of fact, we and others have shown that escalation does not trigger addiction-like behaviour, but is a consequence of its development in vulnerable individuals who also show increased propensity to relapse after forced abstinence (Deroche-Gamonet, Belin & Piazza, Science, 2004; Ducret et al., Bio. Psy. 2015). Additionally, it is difficult, from a psychological perspective, to argue that the dorsolateral striatum is involved in craving as it mediates implicit automatic responses, which, by definition, cannot contribute to the subjective state that is craving, at least in its present state of understanding. 
Again, the purpose of this review is not to delve into these considerations, but we cannot either discuss phenomena we do consider having little of any, relevance with regards to the hypothesis developed here, which is about intrastriatal shifts. 

3) The legend for the hypothetical quadripartite model figure (#3) is too extensive and the text could be curtailed. It might be a good idea to restructure the last part of the section on Astrocytes to discuss the framework for this model by integrative some of the text presented in the legend. 

The legend of the quadripartite model concisely describes each one of the cellular /molecular pathways and each of the description is not more than 1 or 2 sentences, thus we are afraid we cannot curtail the legend as it would then miss out important information. We deliberately refer to each one of these different mechanisms in the figure legend, instead of the manuscript itself, since most of the relevant information has been described in another review (Fouyssac, Everitt & Belin Everitt, 2017). However, the original elements here, namely those pertaining to mechanisms whereby astrocytes respond to dopamine or release glutamate, are also detailed in the core of the manuscript. 


Reviewer: 2 

Comments to the Author 
In this review, M. Fouyssac and D. Belin aim at covering evidence from the literature for a role of striatal astrocytes in the development of addiction. Functional implication of glial cells remains poorly investigated to date and, more so, in the context of drug addiction. This review is thus tackling a very important aspect by addressing the molecular mechanisms underlying the fine-tuning of dopamine signalling as well as its modulation in pathological conditions. However, the content of the manuscript appears very disappointing and the focus remains unclear because a large part of the review relates to the modulation of neuronal connectivity in various models of addiction without any clear link to the intended topic. 
Following a brief introduction raising interest in the functional role of astrocytes, the first section of the review is devoted to the modulation of neuronal connections influencing striatal dopamine level in various behavioural paradigms. Glutamate transporters present in astrocytes are superficially broached and do not bring any real insight. The second part of the review is entirely devoted to the potential therapeutic interest of N-acetylcysteine to prevent relapse and surprisingly concludes on the importance of astrocyte-dependent glutamatergic component on dopaminergic mechanisms in different models related to addiction and the high number of connections one astrocytic cell can provide. Again, the link with the functional role of astrocytes remains elusive. 

We thank this reviewer for this comment. We have restructured the manuscript entirely, and we believe it reads much better now. 
The hypothesis supported in this review is about the role of astrocytes as a cellular node of neural systems adaptations taking place in the striatum over the course of drug addiction. We now start with offering the neural systems framework and we progressively introduce the role of astrocytes in each particular aspect of that framework. 

The last part aims at reviewing the molecular components expressed on astrocytes that may actively contribute to the control of glutamatergic homeostasis and influence dopaminergic signalling. However, this part mixes transporters and GPCRs and would benefit from a better organization of its content. Finally, the authors propose to integrate the astrocytic component in a more comprehensive model of the striatal synapse. However, this model is only presented in the legend of figure 3 and appears somewhat at odd with the content of the main text. 

We have discussed the molecular mechanisms that may contribute to the ability of the striatal astrocytes to orchestrate local and more widespread adaptations within the striatum within functional units. We do not see why there should be a need to discuss GPCRs and transporters separately in this context. 

In addition, no reference to previous literature is included for the already known molecular aspects. 
Beside the absence of a clear guideline, a major drawback of the review is the lack of references suggesting a functional role for astrocytes in drug addiction. See for example Bowers & Kalivas 2003 EJN, Green-Sadan et al 2003 EJN, Nam et al 2013 J Neurosci, Arezoomadan et al 2016 Neurochem Res, Arezoomadan et al 2016 Brain res Bull, Kitamura et al 2010 Leg Med. 

We beg to disagree with this reviewer. Perhaps they consider we did not cite well enough the labs to which they refer here, but there were, and are, many references to the role of astrocytes in drug addiction in this manuscript. Nevertheless, we have added references wherever we thought necessary. 

For the molecular aspects, the authors could also refer to very recent reviews by M.D. Scoffield 2018 Biol Psy on the role of astroglial GPCRs and mechanisms of glutamate release and by Linker et al 2018 EJN on glial mechanisms underlying substance abuse addiction. I would suggest reshaping the current manuscript to provide a complementary, original and focused viewpoint on the functional role of astrocytes in the striatum derived from the integrated perspective of the models and plasticity/relevance of neuroanatomical connections. 

The hypothesis supported in this review aims to bring astrocytes into a neural systems framework focusing on the transitions in the locus of control over behaviour from the ventral to the dorsolateral striatum that occur over the course of drug addiction. Since the scope is not on the state of the art of the molecular adaptations, identified so far almost exclusively in the nucleus accumbens, in the context of CPP, sensitization or extinction/reinstatement procedures, each falling short of triggering adaptations that are at the core of the present hypothesis, we think it would detract the reader to delve into these molecular mechanisms. We actually acknowledge and refer to, the excellent recent reviews that cover these aspects rather exhaustively. 

Other points 
- P12 line 13 :To the best of my knowledge, there is still no evidence for dopamine D1-adenosine A1 heteromers in the striatum. 

Toda and colleagues demonstrated, to the best of our knowledge, for the first time, in vivo, the presence of A1-D1 heteromic receptor complexes in the striatum of the rat (“Repeated cocaine administration changes the function and subcellular distribution of adenosine A1 receptor in the rat nucleus accumbens”, Toda, Alguacil and Kalivas, Journal of Neurochemistry, 2003). 

- The work by Bull et al 2014 is instrumental to the content of the present review to support the role of astrocytes in drug addiction. Therefore, the experimental data supporting this conclusion should be described in more detail. 

We beg to disagree with this reviewer. The elegant work of Bull and colleagues demonstrates a role for astrocytes in alcohol reinforcement and performance under (a not too demanding) progressive ratio schedule of reinforcement. While we believe it is indeed important to refer to that paper, since that study exclusively focused on the accumbens we do not think it is relevant enough to aDLS-dependent drug seeking habits and associated intrastriatal shifts for us to provide a more detailed description of the methods deployed by the authors. Nevertheless, we were very impressed indeed by that study. 

- References Ducret et al 2015 and Hodebourg et al 2018 are incomplete 

“Ducret et al 2015” reference has been amended, however, “Hodebourg et al 2018” has not been included in an issue yet so it can’t be amended as yet. 


- The message and added value of figures 1 and 2 are both unclear in the intended context of the review. 

We believe former figures 1 and 2, now figures 2 and 3 , are actually very important in the context of this review as they provide a unique illustration of the experimental evidence for an involvement of astrocyte -dependent mechanisms, as revealed by the behavioural effects of NAC, on aDLS dopamine-dependent drug seeking habits, which is the focus of the hypothesis defended in the review. We believe that this comes across more clearly having completely reorganized the manuscript.

2^nd^ Editorial Decision	18/03/19

_____________________________________________________________________________________

Beyond drug-induced alteration of glutamate homeostasis, astrocytes may contribute to dopamine-dependent intrastriatal functional shifts that underlie the development of drug addiction. A working hypothesis. 

Dear David, 

Many thanks for the revised version of your manuscript.  It has now been seen by the oiginal reviewers and the editorial team and we are pleased to say that that it will be accepted for publication in EJN following a few minor revisions.   

As you can see below, both reviewers are statisfied by the revisions.  However, Reviewer 2 still raises a couple of concerns.  Please include the references as suggested by the reviewer and Figure 4 is a bit complex.  We see the point about co-localization of D1 and D2 receptors and the issues of heteromers.  Please take a look at it in the light of his/her suggestions.  Note that it will not need to go out to review again.   

When finalised, please upload your complete revised manuscript onto the website as a Word (.doc or .docx), or .rtf file.  Please also ensure that a complete set of tables and figures is included as separate files, even if these have not changed from the originals.  At this stage it is necessary to provide high resolution figures.  Please see important instructions below. 

Please go into https://mc.manuscriptcentral.com/ejn - Author Centre - manuscripts with decisions where you will find a 'create a revision' link under 'actions'. We ask that you please indicate the way in which you have responded to the points raised by the Editors and Reviewers in a letter. Please upload this response letter as a separate Word (.doc) file using the file designation "Authors' Response to Reviewers" when uploading your manuscript files. Please DO NOT submit your revised manuscript as a new one.  Also, please note that only the  Author who submitted the original version of the manuscript should submit a revised version. 

If you are able to respond fully to the points raised, we shall be pleased to receive a revision of your paper within 30 days.

Once again, thank you for submitting your work to EJN and we look forward to seeing the revision. 

Best wishes, 

Paul & John 
co-Editors in Chief, EJN 

Reviews: 

Reviewer: 1 

Comments to the Author 
The revisions made by the authors are satisfactory and I do not have any further concerns. 

Reviewer: 2 

Comments to the Author 
The authors have substantially reorganized the content of the manuscript that now reads more easily. The focus on astrocyte contribution to neurotransmission at the level of the striatal synapse appears more clearly. 
I still have a couple of remarks. Although the authors indicate that they refer to recent reviews that cover the molecular mechanisms and added the reference Scoffield 2018 Biol Psy, I would suggest to also include the review by Linker et al 2018 EJN for extensive coverage of the topic. 
Figure 4 is a little confusing. The authors locate dopamine D1 and D2 receptors on the same MSN whereas such co-localization has only been reported so far in 5% and 20% of the MSNs in the dorsal striatum and nucleus accumbens core respectively (Hasbi et al 2018 Frontiers Pharmacol). Similarly, A2a/D2, A1/D1, A1/D1 and A2a/CB1heteromers are mentioned in figure 4 but others such as MOP/D1, MOP/CB1, H3/D1, D3/D3nf,… have also been identified in the striatum and their expression can be affected by drug intake (for recent reviews see for example Borroto-Escuala et al 2017 Frontiers in Cellular Neurosci; Derouiche & Massotte 2018 Neurosci Biobehav Rev). Although heteromers are undoubtedly participating to drug-induced plasticity, our current knowledge about their role remains fragmentary. D1/D2 and D2/A2a appear to modulate habit formation (He et al 2016 Frontiers Mol Neurosci) and to be involved in reinstatement of cocaine seeking (Hasbi 2018 Frontiers Pharmacol) but the function of the others in these behaviours remains to be determined. Heteromers are not instrumental to the understanding of the proposed model, I would therefore suggest to remove them from the figure (and maybe briefly mention their potential implication) unless the authors wish to comprehensively discuss their contribution to their model.

_____________________________________________________________________________________

Authors’ Response

_____________________________________________________________________________________

Dear Paul and John, 


We would like to thank you and the reviewers for the positive appraisal of the revised version of our manuscript. 
We have included the reference Linker et al., 2018, EJN amongst the recent reviews that have covered the crucial importance of astrocytic mechanisms in the ventral striatum triggered by drug exposure. 
In figure 4, we had deliberately located D1 and D2 receptors onto a unique MSN for the sake of clarity. We have now made an explicit statement about this potential confounding issue in the figure legend 
We agree with reviewer 2 that the precise role of heterodimers such as D1/A1, D2/A2A, A1/A2A or A2A/CB1 receptors, as showed in figure 4, have not been elucidated within the context of drug exposure or drug addiction. Elements in figure 4 are not necessarily restricted to the addiction literature, but instead represent the potential mechanisms that can be aberrantly engaged by drugs of abuse. Thus, the purpose of this figure is to highlight the multiple routes of communication that exist between neurons and astrocytes by which alterations in the dopaminergic system initially hijacked by drugs of abuse may contribute to the drug-induced disruption of glutamate homeostasis. 
Within this framework, we believe that the readers will easily understand that such heterodimers could potentially play a role in the gating of dopaminergic and glutamatergic information orchestrated by astrocytes. 
We believe that we have addressed reviewer 2’s outstanding comments, and we hope that our manuscript will be considered suitable for publication in European Journal of Neuroscience. 

With my best wishes, 

David
